# Mechanisms and Therapeutic Strategies of Viral Myocarditis Targeting Autophagy

**DOI:** 10.3389/fphar.2022.843103

**Published:** 2022-04-11

**Authors:** Kun Yu, Ling Zhou, Yinhui Wang, Chengxin Yu, Ziyi Wang, Hao Liu, Haoran Wei, Liang Han, Jia Cheng, Feng Wang, Dao Wen Wang, Chunxia Zhao

**Affiliations:** ^1^ Division of Cardiology, Departments of Internal Medicine and Hubei Key Laboratory of Genetics and Molecular Mechanisms of Cardiological Disorders, Tongji Hospital, Tongji Medical College, Huazhong University of Science and Technology, Wuhan, China; ^2^ GI Cancer Research Institute, Tongji Hospital, Tongji Medical College, Huazhong University of Science and Technology, Wuhan, China; ^3^ Department of Integrated Traditional Chinese and Western Medicine, Tongji Hospital of Tongji Medical College of Huazhong University of Science and Technology, Wuhan, China

**Keywords:** viral myocarditis, coxsackievirus B3, autophagy, cardiomyocytes, cardiac fibroblasts

## Abstract

Viral myocarditis is caused by infection with viruses or bacteria, including coxsackievirus B3 (CVB3), and is characterized by acute or chronic inflammatory responses in the heart. The mortality associated with severe viral myocarditis is considerable. In some patients, viral myocarditis may develop into dilated cardiomyopathy or heart failure. Autophagy is involved in a wide range of physiological processes, including viral infection and replication. In the present review, we focus on the responses of cardiac tissues, cardiomyocytes, and cardiac fibroblasts to CVB3 infection. Subsequently, the effects of altered autophagy on the development of viral myocarditis are discussed. Finally, this review also examined and assessed the use of several popular autophagy modulating drugs, such as metformin, resveratrol, rapamycin, wortmannin, and 3-methyladenine, as alternative treatment strategies for viral myocarditis.

## 1 Introduction

Viral myocarditis refers to localized or diffuse acute or chronic inflammatory lesions in the myocardium that are caused by viral infection, which could cause serious acute heart damage or chronic persistent heart injury (Rroku et al., 2020). Coxsackievirus B3 (CVB3) is the primary cause of viral myocarditis (Zhang et al., 2020). Studies have shown that autophagy is a highly conserved catabolic process that can affect the prognosis of viral myocarditis by regulating virus replication and release and affecting the immune response and cell apoptosis (Garmaroudi et al., 2015; Sin et al., 2015; Xin et al., 2015). Thus, autophagy is comprehensively involved in the development of viral myocarditis, and it is likely to be a promising therapeutic target for treating viral myocarditis. However, evidence on crosstalk between viral myocarditis and autophagy is not currently adequate. This paper reviews the published studies on autophagy alterations in viral myocarditis and alterations in autophagy in viral myocarditis that impact disease development. Moreover, we discussed the potential mechanisms and future prospects for using autophagy regulators for viral myocarditis adjuvant treatment.

## 2 Epidemiology, Pathogens, Pathogenesis, and Pathology of Viral Myocarditis

Viral myocarditis is a type of infective cardiomyopathy with local or diffusive and acute or chronic inflammatory myocardial lesions caused by viral infection (Du et al., 2020). This condition has an incidence rate of 10–22 per 100,000 individuals (Olejniczak et al., 2020). Viral myocarditis mainly occurs in young and middle-aged patients, accounting for 12% of sudden deaths in patients less than 40 years of age (Blauwet and Cooper, 2010; Olejniczak et al., 2020).

**FIGURE 1:**
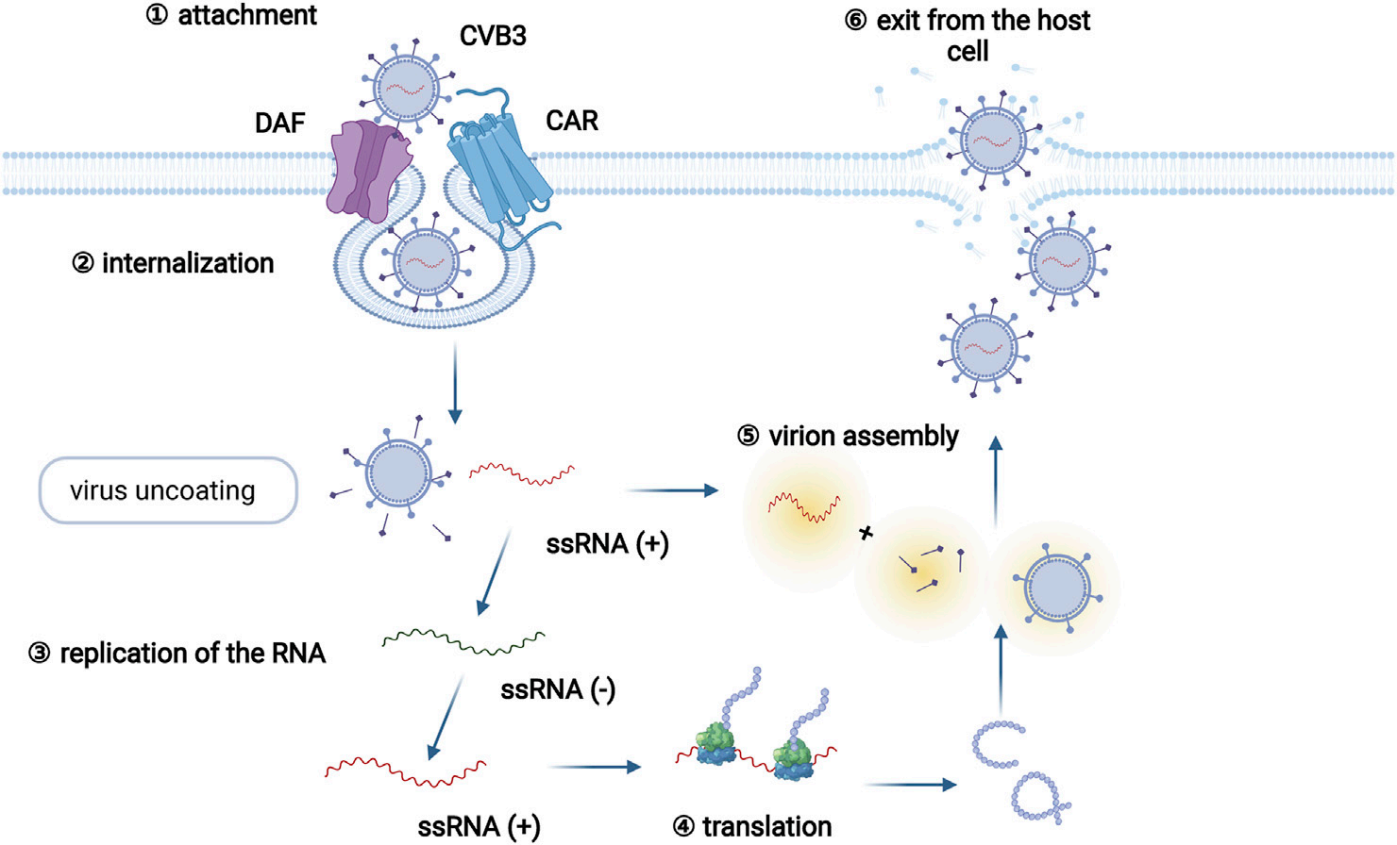
The CVB3 life cycle. The CVB3 life cycle mainly involves six different stages: viral attachment to the host cell, penetration, RNA genome replication, viral protein translation, assembly of virions, and exit from the host cell. Specifically, CVB3 infection begins by the coupling of decay-accelerating factor (DAF, primary attachment protein or coreceptor) and coxsackievirus and adenovirus receptor (CAR, internalization receptor) on host cells. Virus binding to CAR facilitates virus internalization. Following internalization and virus uncoating, the positive-sense RNA of CVB3 enters the cytoplasm and serves as a template for the translation of viral polyprotein. Subsequently, the newly synthesized polyprotein is cleaved into several structural and functional proteins. Together with the positive RNA strands, new virions are assembled and released as progeny viruses to initiate new rounds of infection.

A series of viruses, including enterovirus, adenoviruses, human parvovirus, and human herpesviruses, are able to induce myocarditis (Blauwet and Cooper, 2010). Coxsackievirus B3 (CVB3), which belongs to the enterovirus genus, is the most common pathogen among patients with viral myocarditis, and infects approximately 25% of viral myocarditis patients (Pollack et al., 2015; Błyszczuk, 2019). The life cycle of CVB3 mainly includes viral attachment to the host cell, penetration, RNA genome replication, viral protein translation, virion assembly, and exit from the host cell (Zaragoza et al., 1997) (Figure 1). In addition, the infection pattern of CVB3 has been adequately studied in animal models of myocarditis, and many cell and animal studies on viral myocarditis have focused on CVB3 (Pollack et al., 2015; Błyszczuk, 2019).

The process of CVB3 infection in myocarditis can be divided into three stages. In the first stage, cardiomyocyte structure is directly destroyed by CVB3, which in turn facilitates viral entry into cells (Maekawa et al., 2007). However, this stage is easy to ignore because the initial damage caused by CVB3 can be partly blocked by innate immune cells (McCarthy et al., 2000). Then, CVB3 replicates and leads to a cascade of cytokine release during the second stage, which activates adaptive immune responses. During this period, autoimmune-mediated damage has detrimental effects on the heart (Dennert et al., 2008). T cell infiltration typically peaks at 7 d after infection, which is often the most severe stage of viral myocarditis (Badorff and Knowlton, 2004). The pathological manifestations of these two stages can be summarized as follows: Degeneration or necrosis occurs in individual cardiomyocytes or a small cluster of myocardial fibers during the acute phase. The infiltration of monocytes, lymphocytes, and plasma cells can be observed around myocardial interstices and small vessels with accompanying low levels of neutrophils and eosinophils (Aretz, 1987).

With decreasing viral titers, the inflammatory response gradually subsides. Subsequently, some patients exhibit dilated cardiomyopathy and other sequelae due to a chronic inflammatory response in the myocardium and myocardial injury, which is the third stage of viral myocarditis (Mason, 2003). During this stage, autoantibodies that cross-react with self-antigens persist in the viral genome (Caforio et al., 2002). Autoantibodies are generated, possibly because the intracellular proteins of damaged cardiomyocytes are exposed to the immune system (Shioi et al., 1996). Then, T cells are activated and produce cytokines and matrix-degrading proteinases, which induce persistent myocardial injury and reparative myocardial fibrosis (Shioi et al., 1996; Pauschinger et al., 2004; Heymans, 2006; Heymans et al., 2006). These events collectively lead to the formation of granulation tissue and interstitial fibrosis, which is mainly observed around muscle fascicles, small vessels, and the endocardium. There may be a scattered small area of scars, cardiomyocyte compensatory hypertrophy, and degenerative calcification (Aretz, 1987).

## 3 The Autophagy Process

Autophagy is a highly conserved intracellular degradation and recycling pathway in eukaryotes (Shibutani et al., 2015). By degrading useless cytosolic contents in lysosomes, autophagy provides metabolic substrates and energy for cells. As a widespread metabolic pathway, autophagy plays vital roles in maintaining cellular homeostasis (Menzies et al., 2015). Autophagy can be divided into three categories in mammalian cells: macroautophagy, microautophagy, and chaperone-mediated autophagy (CAM) (Parzych and Klionsky, 2014). Macroautophagy starts with the synthesis of autophagic vesicles with a double-layer membrane structure. Then, the vesicles, also called autophagosomes, fuse with lysosomes to form autolysosomes, resulting in content degradation (Li et al., 2014a). During the process of microautophagy, contents near lysosomes that are targeted for degradation will be engulfed by lysosomal membrane invagination (Mijaljica et al., 2011). CAM is the third form of autophagy. After being recognized by molecular chaperones, cytoplasmic proteins with specific pentapeptide motifs bind to specific receptors on the lysosomal membrane and enter lysosomes for degradation (Massey et al., 2004).

**FIGURE 2:**
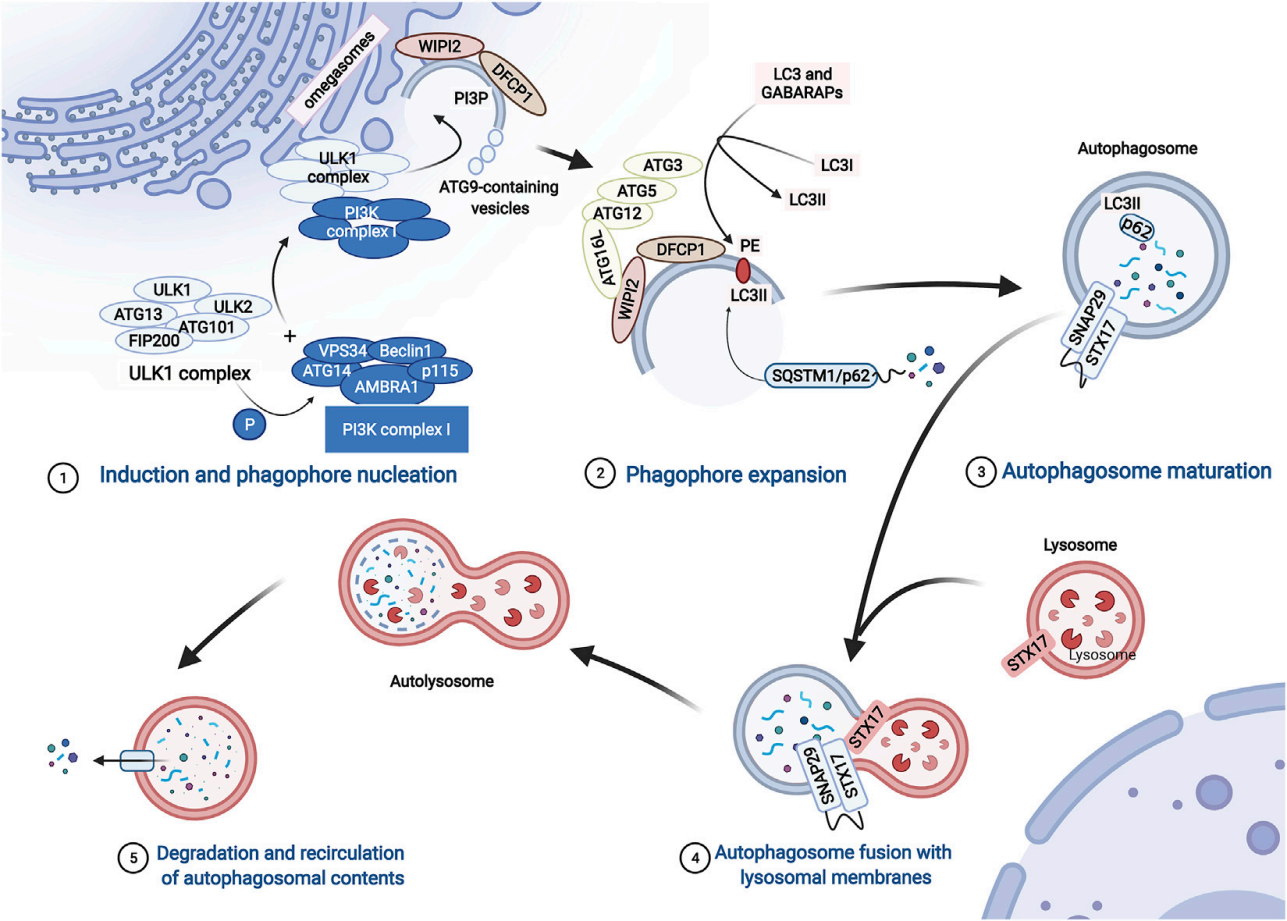
The process of autophagy. The process of autophagy includes induction and phagophore nucleation, phagophore expansion, autophagosome maturation, autophagosome fusion with lysosomal membranes, degradation, and recirculation of autophagosomal contents. Induction and phagophore nucleation occur in the structure known as the “omegasome” on the endoplasmic reticulum membrane and are mainly mediated by the ULK1 complex and PI3KC3 complex I. Local activated PI3P and recruited Atg9 provide the raw materials required for membrane formation. Then, the ATG12-ATG5-ATG16L1 complex is recruited to the membrane of the phagophore, enhancing the conversion of LC3 and GABARAPs to membrane-resident PE, promoting phagophore expansion. During the formation of autophagosomes, p62 serves as a bridge connecting LC3 with the ubiquitinated substrate. Finally, the ATG12-ATG5 and ATG8/LC3 conjugation systems mediate the process by which the isolation membrane of the autophagosome forms a closed bilayer membrane structure and matures. Mediated by autophagosome-localized STX17, SNAP29, and lysosome-localized VAMP8, mature autophagosomes fuse with lysosomes to form autolysosomes, which degrade cytoplasmic contents with lysosomal proteases.

The molecular mechanism of macroautophagy (hereafter referred to as autophagy) is complicated and finely regulated. Autophagy is activated under stress conditions, such as starvation or oxidative stress (Mazure and Pouysségur, 2010; Sciarretta et al., 2018). At the beginning of autophagy, ATG13 (autophagy-related protein 13) anchors ULK1 to the preautophagosomal structure (PAS), which is accompanied by the formation of the ULK1 complex that consists of ULK1, ULK2, ATG13, FIP200, and ATG101. Then, the PI3K complex, ATG9A system, ATG12-conjugation system, and LC3-conjugation system are targeted to the PAS and participate in the assembly and formation of autophagosomes in a graded manner (Dikic and Elazar, 2018; Li et al., 2020) (Figure 2).

The ULK1 complex phosphorylates class III phosphatidylinositol 3-kinase complex I (PI3KC3-C1), which consists of VPS34, Beclin1, ATG14, activating molecule in Beclin1-regulated autophagy protein 1 (AMBRA1), and general vesicular transport factor (p115), which in turn activates local phosphatidylinositol-3-phosphate (PI3P) production. Subsequently, PI3P recruits WD repeat domain phosphoinositide-interacting proteins (WIPIs; here WIPI2) and zinc-finger FYVE domain-containing protein 1 (DFCP1). Moreover, ATG9-containing vesicles generated by the secretory pathway are recruited to the PAS, contributing to membrane expansion by delivering additional lipids and proteins. Then, WIPI2 binds to ATG16L1 directly, leading to the recruitment of the ATG12-ATG5-ATG16L1 complex, which enhances the conjugation of ATG8 family proteins (ATG8s) mediated by ATG3. ATG8s include microtubule-associated protein light chain 3 (LC3) and γ-aminobutyric acid receptor-associated proteins (GABARAPs), which are converted to membrane-resident phosphatidylethanolamine (PE) and form membrane-bound lipidated forms. In this conjugation reaction, LC3I is converted to LC3-II, which is a characteristic of autophagic vesicle membranes. Finally, mediated by ATG12-ATG5 and ATG8/LC3) conjugation systems, the isolation membrane of the autophagosome forms a closed bilayer membrane structure and matures (Dikic and Elazar, 2018; Li et al., 2020) (Figure 2).

During the formation of autophagosomes, autophagy receptors serve as a bridge connecting LC3 with the ubiquitinated and non-ubiquitinated substrates (Figure 2). SQSTM1/p62, an important autophagy receptor containing the ubiquitin binding domain, is widely used as a marker to monitor autophagic flux (Mohamud et al., 2018). p62 contains multiple functional domains, including the TRAF6 (tumor necrosis-associated factor 6) binding (TB) domain, microtubule-associated protein 1 light chain 3 (LC3)-interacting region (LIR), and C-terminal ubiquitin association (UBA) domain. Through interactions with these important domains, p62 targets ubiquitinated protein aggregates for autophagic degradation (Shi et al., 2013).

Mature autophagosomes are transferred into the perinuclear region (Garmaroudi et al., 2015). Then, autophagosomes and lysosomes fuse to form autolysosomes. This last step is mediated by soluble N-ethylmaleimide-sensitive factor attachment protein receptor (SNARE) proteins, including autophagosome-localized SNARE protein syntaxin 17 (STX17), synaptosomal-associated protein 29 (SNAP29), and lysosome-localized vesicle-associated membrane protein 8 (VAMP8). Then, many enzymes in the lysosomes degrade the inner membranes of autolysosomes, organelles, and proteins for recycling (Tian et al., 2021) (Figure 2).

## 4 Autophagy in Viral Myocarditis

### 4.1 Influence and Mechanism of Altered Autophagy in Viral Myocarditis

Autophagy is a highly dynamic and multistep process (Lee et al., 2020). The level of autophagy over a certain period of time is called autophagic flux, which is mainly associated with the strength of autophagy induction and the rate at which autophagosomes are fused and digested by lysosomes (Zhang et al., 2013). Previous studies on autophagy evaluated the level of autophagy by measuring the number of autophagosomes. Accurate and comprehensive evaluation of autophagy includes not only the detection of autophagosomes but also the dynamic observation of whether the process of autophagic flux is fluent.

**FIGURE 3:**
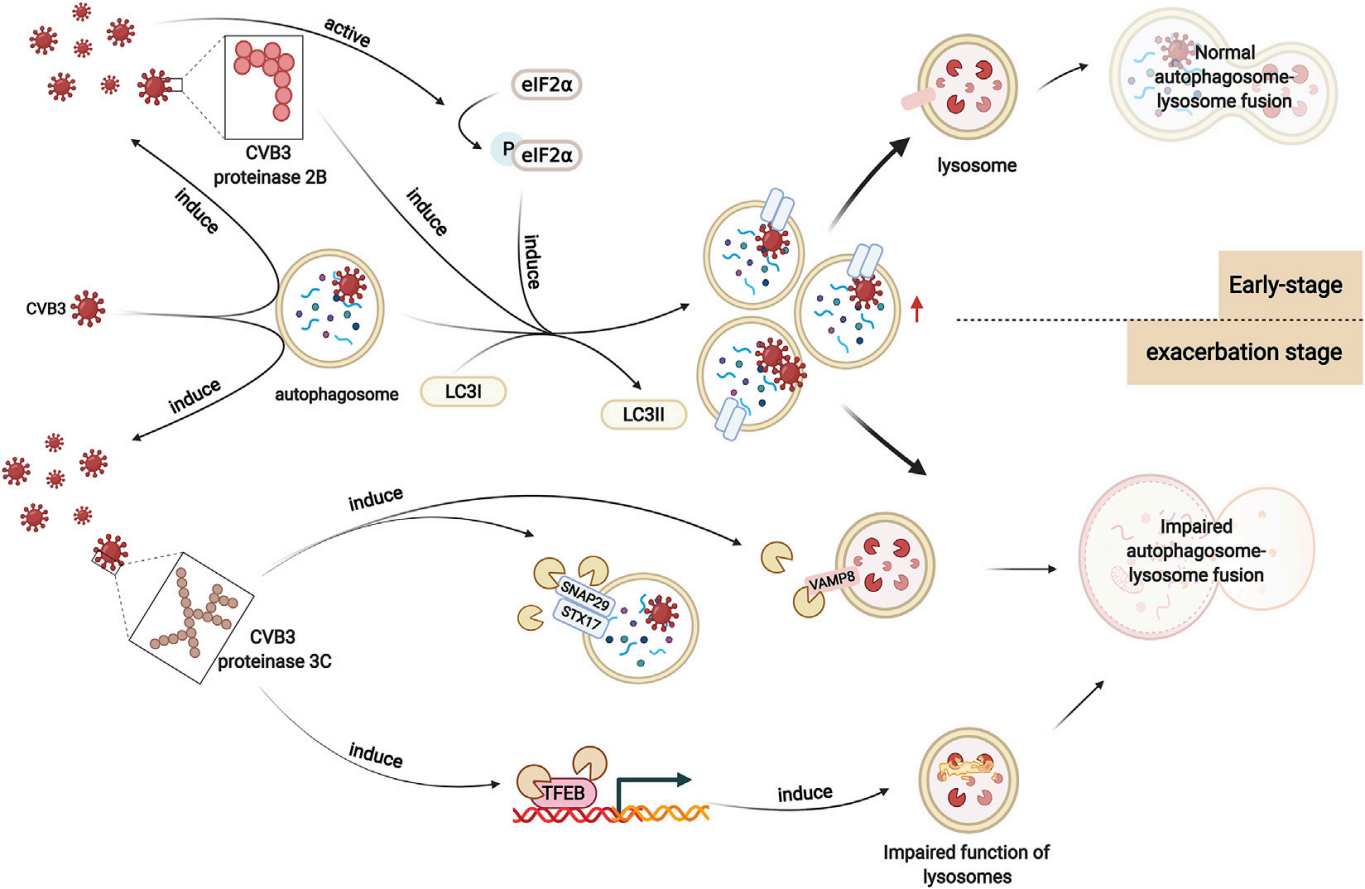
Altered autophagy in viral myocarditis. In viral myocarditis, CVB3 exploits the double-membrane structure of autophagosomes to replicate. CVB3 promotes the formation of autophagosomes in host cells, which may be associated with enhanced eIF2α phosphorylation. CVB3 proteinase 2B also plays a significant role in this process. After CVB3 infection, autophagy is activated. In the early stage, CVB3 is sequestered by autophagosomes and delivered to lysosomes, activating the autophagy positive-feedback pathway. Autophagosomes are subsequently degraded. However, as infection worsens, CVB3 proteinase 3C contributes to the cleavage of autophagy receptors that play a vital role in autophagosome-lysosome fusion. Moreover, CVB3 also targets TFEB for proteolytic processing by CVB3 proteinase 3C to disrupt host lysosomal function. Autophagosome-lysosome fusion is impaired, which affects the degradation of autophagosomes, enhancing viral infection.

Numerous experiments suggest that viral infection results in the alteration of autophagy levels. The number of autophagic vesicles, the expression of LC3I and LC3II, and the ratio of LC3II/LC3I were elevated in heart tissues after CVB3 infection, indicating that autophagosome formation increased (Zhai et al., 2015). The mechanisms by which CVB3 infection promotes autophagosome formation and inhibits autophagosome degradation in viral myocarditis are still unclear. It has been demonstrated that autophagosome formation is increased in CVB3-infected HeLa cells, which is associated with enhanced eIF2α phosphorylation (Wong et al., 2008). Phosphorylated eIF2α can mediate polyglutamine-induced LC3 conversion, which is an essential step in autophagosome formation (Tallóczy et al., 2002; Kouroku et al., 2007). Moreover, CVB3 proteinase 2B, one of the nonstructural proteins of CVB3, can induce autophagy. Expression of 2B alone is sufficient to induce the formation of autophagosomes in HeLa cells. Instead, after the mutation of valine at position 56 in the transmembrane sequences of CVB3 proteinase 2B, CVB3 loses its ability to induce autophagosomes (Wu et al., 2016) (Figure 3).

As described previously, the autophagy adapter protein p62 is widely used to monitor autophagic flux. Zhai et al. found that the level of p62 did not change in CVB3-infected cardiac tissue, which indicated that autophagosomes were not degraded by lysosomes. In the study by Gu et al., the expression level of p62 was decreased in cardiac tissue in CVB3-infected viral myocarditis (Gu et al., 2020). Another study indicated that the p62 level showed an initial decrease but subsequent increase in CVB3-infected HeLa cells, which suggested that there may be a similar trend in autophagosomes in CVB3-infected cardiac tissues (Chang et al., 2017). This finding could be interpreted as the different effects of viral infection on lysosomes at different times.

We hypothesize that in the early stage of infection, CVB3 particles are sequestered by autophagosomes and delivered to lysosomes, activating the autophagy positive-feedback pathway. Autophagosomes are subsequently degraded, resulting in decreased p62 levels (Alirezaei et al., 2012). Moreover, it has been reported that p62 can be cleaved by the CVB3-encoded proteinase 2A at glycine 241 in the TB domain, which may also contribute to a decrease in p62 in viral myocarditis (Shi et al., 2013). In fact, p62 can recognize and immunoprecipitate the viral capsid protein VP1 after infection with CVB3, and knockdown of SQSTM1 leads to enhanced expression of VP1 and increased CVB3 viral titers. Moreover, the binding affinity of VP1 for the cleavage fragments of p62 is markedly decreased, which indicates that p62 cleavage may serve as a strategy by which CVB3 counteracts the role of p62 in viral clearance (Mohamud et al., 2019).

However, the function of lysosomes is disrupted by CVB3 as infection worsens, causing impaired degradation of autophagosomes and increased p62 levels. It has been reported that the SNARE complex-mediated fusion process plays an important role in autophagosome-lysosome fusion (Tian et al., 2021). Upon autophagic stimulation in HeLa cells, STX17 is specifically recruited to mature autophagosomes. Then, STX17 interacts with SNAP29 and VAMP8, driving the fusion of autophagosomes with lysosomes/endosomes. After infection with CVB3, the protein levels of these three SNARE proteins decreases. SNAP29 shows the highest reduction, suggesting the possible cleavage of SNAP29. Cleavage assays *in vivo* (in cells transfected with proteinase constructs) and *in vitro* (using recombinant purified proteinases) identify that the proteinase 3C contributes to this cleavage. SNAP29 is cleaved after the glutamine [Q] residue at position 161, separating its N-terminal coiled-coil domain from the C-terminal SNARE motif. Together with the two SNARE proteins STX17 and VAMP8, the STX17-SNAP29-VAMP8 complex is disrupted, indicating that CVB3 reduces autophagic flux by disrupting the formation of SNARE complexes (Mohamud et al., 2018) (Figure 3).

Moreover, another study on HeLa cells revealed that transcription factor EB (TFEB) is a primary regulator of autophagy and lysosome biogenesis and acts as a novel target of CVB3 proteinase 3C. Specifically, CVB3 proteinase 3C degrades TFEB to TFEB [Δ60], disrupting the function of lysosomes. Autophagic flux is blocked, resulting in the accumulation of autophagosomes and enhancing viral replication (Mohamud et al., 2021) (Figure 3).

In summary, it seems that CVB3 promotes autophagosome formation but inhibit its degradation. However, the mechanism has not been validated in CVB3-infected viral myocarditis, and further mechanistic studies are required in the future.

#### 4.1.1 Altered Autophagy and its Mechanism in Cardiomyocytes in the Context of Viral Myocarditis

Many kinds of cardiomyocytes, including primary cardiomyocytes from SD suckling rats or healthy BALB/c mice and cardiomyocyte cell lines such as H9C2 or HL-1 cells have been infected with CVB3 and used to research alterations in autophagy in viral myocarditis. CVB3 infection *in vitro* activates autophagic signaling in these cells: the level of LC3-II or LC3-II/LC3I increases after CVB3 infection, accompanied by elevated viral RNA, and protein levels. The same effect was observed in cardiomyocytes isolated from BALB/c mice after CVB3 infection *in vivo*. Moreover, by using the autophagy inhibitor 3-methyladenine (3-MA), the CVB3 titer decreased significantly in primary cardiomyocytes from healthy BALB/c mice, H9C2 cells, and HL-1 cells. These results indicate that CVB3 may exploit autophagosomes to facilitate its own replication (Tabor-Godwin et al., 2012; Li et al., 2014b; Zhai et al., 2015; Meng et al., 2020).

**FIGURE 4:**
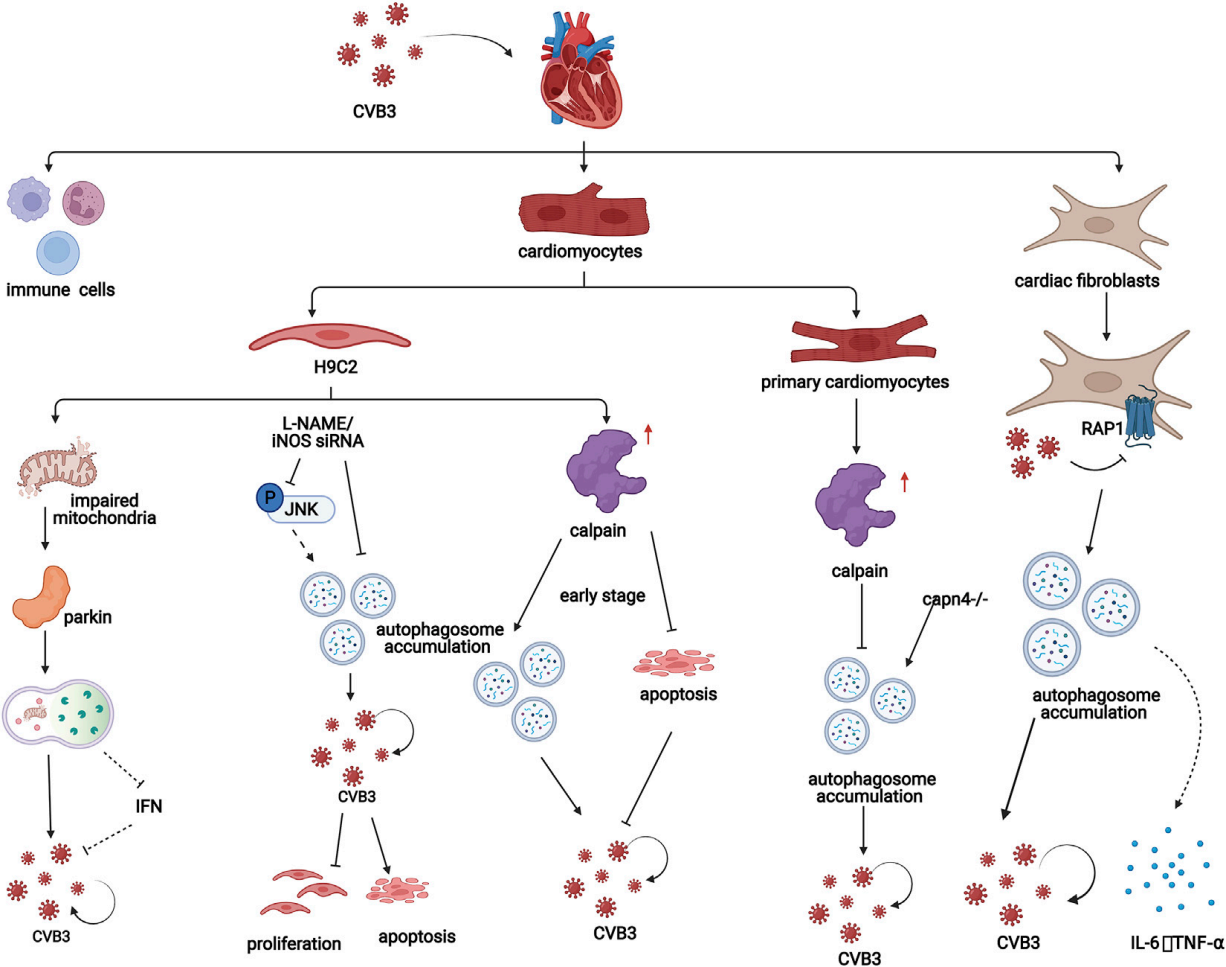
In viral myocarditis, CVB3 influences the process of autophagy in both cardiomyocytes and cardiac fibroblasts, which promotes disease progression through a variety of mechanisms. In H9C2 cells, mitochondrial structures are destroyed by CVB3, which induces mitochondrial autophagy in a Parkin-dependent manner. Additionally, iNOS inhibition attenuates CVB3-induced autophagy activation, increasing cell proliferation, and suppressing cell apoptosis. Moreover, in the early stage of CVB3 infection in H9C2 cells, calpain is beneficial for viral replication by promoting the formation of autophagosomes and inhibiting apoptosis. However, in a study of cardiomyocytes from CVB3-infected suckling mice, inhibiting calpain promoted the formation of autophagosomes and CVB3 replication. In fibroblasts, CVB3 promotes the formation of autophagosomes by inhibiting PAR1 and subsequently contributes to the replication of CVB3 and the release of inflammatory factors.

Calpain may a play crucial role in autophagy in CVB3-infected cardiomyocytes. CVB3 infection also increases the level of calpain. In the early stage of CVB3 infection in H9C2 cells, calpain is beneficial for viral replication by promoting the formation of autophagosomes and inhibiting apoptosis in host cells (Li et al., 2014b). However, in a study using primary cardiomyocytes from CVB3-infected suckling mice, inhibiting calpain promoted the formation of autophagosomes and CVB3 replication. Additionally, inhibiting calpain exacerbates CVB3-induced viral myocarditis *in vivo* (Meng et al., 2020) (Figure 4). These studies indicate that calpain plays different roles in autophagy in CVB3-infected cardiomyocytes, which can be interpreted as the different stages of viral myocarditis and should be researched in the future.

In addition, Li et al. found that in the CVB3-infected H9C2 cardiomyogenic lineage, inhibiting inducible NO synthase (iNOS) could attenuate the formation but promote the degradation of autophagosomes by inhibiting the JNK pathway. The autophagosome membrane, which is required for CVB3 replication, is reduced and iNOS inhibition increases cell proliferation and suppresses cell apoptosis in CVB3-infected H9C2 cells, which provides a new therapeutic strategy for the treatment of CVB3-induced myocarditis (Qi et al., 2017) (Figure 4).

Additionally, CVB3 infection can damage the mitochondrial structure of H9C2 cardiomyocyte lineages. The loss of mitochondrial membrane potential impairs mitochondrial function and induces mitophagy. In this process, reduction in mitochondrial membrane potential can induce stabilization of PINK1 and further facilitate the Parkin migration to the mitochondria which is PINK1-dependent (Vives-Bauza et al., 2010). PINK1/Parkin-mediated pathway can amplify ubiquitin signals on damaged mitochondria. Then damaged mitochondria are enclosed by autophagic membranes and delivered to the lysosomes for degradation (Yamano et al., 2020). Parkin puncta formation and colocalization with LC3-RFP were observed in the mitochondria of CVB3-infected cells, indicating that mitochondrial autophagy induced by CVB3 depends on Parkin. Moreover, carbonyl cyanide m-chlorophenyl hydrazone (CCCP), a mitochondrial autophagy agonist, can promote CVB3 replication in cardiomyocytes by inhibiting interferon (IFN) expression through inhibiting the interaction between mitochondrial antiviral signaling protein (MAVS) and TANK-binding kinase 1 (TBK1) (Oh et al., 2021) (Figure 4).

#### 4.1.2 Altered Autophagy and its Mechanism in Cardiac Fibroblasts in the Context of Viral Myocarditis

Cardiac fibroblasts constitute approximately 27% of total heart cells in mice and approximately 64% in rats, and these cells play important roles in the repair of cardiac pathological injury and compensation of cardiac dysfunction (Deb and Ubil, 2014; Shinde and Frangogiannis, 2014). It has been shown that the replication capacity of CVB3 is stronger in cardiac fibroblasts than in other cardiomyocytes (Lindner et al., 2014). Moreover, the degradation speed of CVB3-infected cardiac fibroblasts is slow, and fibroblasts can develop into a stage of persistent viral replication (carrier state). Therefore, cardiac fibroblasts may play crucial roles in CVB3-induced myocarditis (Heim et al., 2000). An increased number of double-membrane autophagosome-like vesicles, increased LC3 punctate distribution, and elevated LC3-II levels were observed in fibroblasts derived from the hearts of CVB3-infected BALB/c mice, which indicates that CVB3 replication can induce the formation of autophagosomes in cardiac fibroblasts *in vitro* and *in vivo* (Zhai et al., 2016). Furthermore, by introducing pMKS1, which contains the full-length cDNA of CVB3, into cardiac fibroblasts, Zhai et al. found that the distribution of LC3 fluorescence was punctate in cardiac fibroblasts transfected with pMKS1, which is similar to that in fibroblasts infected with CVB3, indicating that autophagosome formation is the result of CVB3 replication (Zhai et al., 2016).

Protease-activated receptor 1 (PAR1) is widely expressed in humans and mice. This factor contributes to the innate immune response to viral infection. Bode et al. showed that compared with wild-type controls, mice lacking PAR1 in cardiac fibroblasts had exacerbated CVB3-induced myocarditis with increased CVB3 genomes, inflammatory infiltrates, inflammatory mediators, and macrophages in the heart (Bode et al., 2021). PAR1 activation is associated with increased numbers of autophagosomes in CVB3-infected murine embryonic fibroblasts (MEFs). In addition, decreasing the level of PAR1 exacerbates CVB3 replication in MEFs. MEFs that were not stimulated with CVB3 or other factors were able to release PAR1-activating proteases. Therefore, autophagy can be inhibited in an autocrine and/or paracrine manner in a PAR1-dependent manner, which indicates that CVB3 infection may inhibit autophagy by activating PAR1 (Weithauser et al., 2016) (Figure 4).

In addition, CVB3 can transiently induce the production of cytokines in cardiac fibroblasts. It has been reported that CVB3 replication increases the production of IL-6 and TNF-α in cardiac fibroblasts, which might be related to autophagy activation (Heim et al., 2000) (Figure 4). A study of fibroblasts derived from CVB3-infected mouse ventricles treated with 3-MA or siRNA targeting Atg7 showed that inhibiting autophagy played a protective role in the mouse heart by downregulating the expression of the proinflammatory cytokines IL-6 and TNF-α (Cheng et al., 2014; Zhai et al., 2016).

Moreover, it is known that autophagy is involved in innate immunity and adaptive immunity via pathogen degradation and antigen presentation, promoting the production of IFN, which is important in protecting against viral infection (Schmid and Münz, 2007). IFN receptor-knockout mice are sensitive to CVB3 infection, which can lead to the deterioration of myocarditis and increased mortality (Althof et al., 2014). However, no significant changes in the expression of IFN in cardiac fibroblasts were observed after CVB3 infection. This finding can be interpreted as the result of CVB3 proteinase 3C cleaving MAVS, which is an adaptive molecule by which the virus evades host immune responses. These findings indicate that autophagy in cardiac fibroblasts plays a vital role in the mechanisms of CVB3-induced myocarditis. Cardiac fibroblasts not only promote viral replication but are also involved in inflammatory responses by secreting inflammatory cytokines, which is likely related to autophagy (Zhai et al., 2016).

### 4.2 Effect of Altered Autophagy on the Development of Viral Myocarditis

Autophagosome formation is increased in CVB3-infected cardiac tissues, but the fusion of autophagosomes and lysosomes is blocked (Tian et al., 2018). Considering that autophagosomes are involved in the regulation of many important physiological processes, changes in autophagosome levels may also influence the development of viral myocarditis. Recent studies have shown that autophagy may influence the development of myocarditis by inhibiting viral replication, influencing cell apoptosis and inducing cellular immune responses (Luo et al., 2010; Yoshida, 2017; Ho et al., 2021).

#### 4.2.1 Autophagy Promotes the Replication and Release of CVB3 in Viral Myocarditis

CVB3 can promote the development of myocarditis by directly inducing apoptosis in cardiomyocytes in a murine model of viral myocarditis. Studies indicate that double membrane autophagosomes in the host can be a site of viral RNA synthesis by recruiting polysomes and promote the assembly of the viral replication complex (Wong et al., 2008). Studies using HeLa and HEK293T cells further demonstrated this finding. It has shown that autophagosome formation is increased in CVB3-infected cells, accompanied by increasing levels of CVB3 viral protein 1 (VP1). Knocking down ATG7, a crucial protein in autophagosome formation, alleviates the states of host cells. Similarly, VP1 levels in host cells are decreased after knocking down Beclin-1 or VPS34, which are two important proteins in autophagosome formation, or treating cells with the autophagosome formation inhibitor 3-MA. In contrast, treatment with the autophagy agonist rapamycin increases VP1 levels in host cells. The fusion of autophagosomes and lysosomes is the degradation pathway of autophagosomes. Decreasing the degradation of autophagosomes also leads to the accumulation of autophagosomes (Wong et al., 2008). It has been confirmed that the lack of LAMP-2 impairs the fusion of autophagosomes and lysosomes so that the degradation of autophagosomes is inhibited. In addition, in LAMP-2-knockdown cells, the VP1 level in host cells is increased, and more virus particles are released. These results suggest that double-membrane autophagosomes may promote CVB3 replication in viral myocarditis (Tanaka et al., 2000).

In addition to increasing viral replication, autophagosomes may also promote host cell release of CVB3. Robinson et al. constructed fluorescent timer protein-tagged CVB3 (Timer-CVB3), to distinguish different infection times in host cells. The study showed that extracellular membrane vesicles (EMVs), which contained viral proteins, were shed from the host cells after Timer-CVB3 infection. There are viruses in EMVs, accompanied by high LC3II levels. A possible explanation for this is that double-membraned autophagosomes are the source of EMVs, and autophagosomes can fuse with the cell membrane, resulting in single-membrane EMV release. This finding indicates that autophagosomes may promote viral release without cell lysis by releasing EMVs (Robinson et al., 2014). Unfortunately, the increase in autophagosome formation and inhibition of autophagosome and lysosome fusion have not been validated in a viral myocarditis model.

#### 4.2.2 Impairment of the Autophagy–Lysosome Pathway Exacerbates CVB3 Infection-Induced Apoptosis

*In vivo* studies of CVB3-induced viral myocarditis indicate that CVB3 can trigger endoplasmic reticulum stress (ERS), resulting in the accumulation of many unfolded or misfolded proteins in the endoplasmic reticulum (Cai et al., 2015). Normally, the autophagy-lysosomal pathway is induced to degrade unfolded or misfolded proteins when they cannot be completely cleared by the proteasome-mediated degradation pathway. However, CVB3 infection impairs the autophagy-lysosomal pathway so that unfolded or misfolded proteins cannot be degraded properly. Finally, the accumulated unfolded or misfolded proteins induce apoptosis via the unfolded protein response (UPR) and C/EBP homologous protein (CHOP) (Yoshida, 2017).

#### 4.2.3 Implications of Autophagy in the Immune System in Viral Myocarditis

The local immune environment in cardiac tissues in viral myocarditis profoundly affects the progression of viral myocarditis, which can be divided into three main phases. The first phase is viral entry and innate immune system activation, the second phase is adaptive immune response activation, and the third phase is recovery or disease progression (Ho et al., 2021).

Within 1–7 d post-infection, the innate immune system is activated by CVB3. Cardiac toll-like receptors (TLRs) recognize general infection patterns and release proinflammatory cytokines such as interleukin-1β (IL-1β), IL-6, IL-18, TNF-α, and type I and type II interferons (IFNs). Then, the activation of TLR 3, TLR 4, and TLR 9 by pathogen-associated molecular patterns (PAMPs) during viral myocarditis seems to worsen the severity of inflammation in CVB3-infected mouse hearts. Innate immune cells, including natural killer cells (NK cells), natural killer T cells (NKT cells), monocytes, macrophages, and dendritic cells, are recruited to the infected area by these cytokines. It is thought that the presence of NK and NKT cells is beneficial during the early phase of viral myocarditis, while other cells of the innate immune system appear to have a negative effect (Ho et al., 2021).

In the second phase, the adaptive immune system responds to CVB3 infection. The most severe tissue damage occurs in this phase due to high viral replication and inflammatory processes. There is increased release of the cytokines TNF, IL-1a, IL-1β, IL-2, and IFN-γ, as well as the recruitment of virus-specific T- and B-lymphocytes, accompanied by cellular and humoral antibodies to resist the infection (Ho et al., 2021). Pathogen neutralization during the adaptive response is more complex than neutralization by the innate immune system, and its contribution to cardiac injury is more sophisticated (Corsten et al., 2012). There is strong evidence that autoimmune reactivity during CVB3-induced inflammation contributes significantly to disease severity (Huber, 2006; Rose, 2009). Moreover, compared with Th2 signaling, which favors antibody-mediated immune responses, Th1 signaling, which favors IFN-γ-mediated cellular immune responses, seems to exacerbate autoimmune cardiac damage (Huber and Pfaeffle, 1994).

In the third phase, the disease develops toward recovery and viral clearance or toward irreversible chronic DMC. Approximately 60–70% of myocarditis patients recover from infection because of an intact immune system (Dominguez et al., 2016).

Autophagy is closely associated with innate immune responses mediated by pattern-recognition receptors, including Toll-like receptor (TLR), NOD-like receptor, and RIG-like receptor (RLR) (Lee et al., 2007; Tal and Iwasaki, 2009; Tormo et al., 2009). In addition, autophagy profoundly impacts the differentiation and function of immune cells by modulating essential cellular physiology. While evidence that autophagy is an important pathway for antiviral immune responses, very little is known about autophagy in antiviral defense during CVB3 infections in myocarditis (Lee and Iwasaki, 2008). As previously described, the innate antiviral response is mediated at least in part by TLR3. The absence of TLR3 significantly increases mortality after infection with enteroviruses that cause myocarditis in mice. Moreover, CVB3 infection of cell lines with TLR3 mutations abrogates type I interferon pathway activation, leading to increased viral replication. Type I interferon signaling is mediated by TLR3 and requires cellular autophagy since this process can be suppressed by bafilomycin A1 and 3-methyladenine, which are inhibitors of lysosomal proteolysis, and reduced expression of Atg5, Beclin1, or microtubule-associated protein 1 light chain 3β (MAP1LC3β) (Gorbea et al., 2010).

In addition, among T cells, the differentiation of CD8^+^ T cells relies on high autophagy levels (Kovacs et al., 2012; Sowell et al., 2014). In contrast, helper T cell differentiation is supported by a relatively lower autophagy level (Delgoffe et al., 2011). Interestingly, regulatory T cell (Treg) stability requires a certain degree of autophagy, and higher or lower mTORC1 impairs the immunosuppressive function of Tregs (Zeng et al., 2013; Wei et al., 2016). Plasma cells exert dual effects on viral myocarditis. On the one hand, humoral immune responses mediated by plasma cells are helpful for viral clearance. On the other hand, autoantibodies produced by plasma cells also result in long-term chronic cardiac injury. Notably, autophagosomes are required for immunoglobulin production in plasma cells (Pengo et al., 2013). In addition, the polarization of macrophages is influenced by autophagy. Inhibiting autophagy promotes M1-polarized macrophages, and promoting autophagy promotes M2-polarized macrophages (Liu et al., 2015). Autophagy is essential for inflammation mediated by neutrophils and neutrophil extracellular traps (NETs) (Itakura and McCarty, 2013; Bhattacharya et al., 2015). However, how autophagy influences these immune responses in CVB3-induced viral myocarditis is still unclear and should be a focus of future research.

## 5 Potential Therapeutic Effect of Treatments Associated With Autophagy in Viral Myocarditis

### 5.1 Application of Autophagy Agonists in Viral Myocarditis

In viral myocarditis, infection with CVB3 blocks autophagic flux in host cells, leading to the accumulation of damaged organelles and misfolded proteins, resulting in disease development. It has been reported that activation of the AMPK signaling pathway ameliorates autophagic flux (Han et al., 2016). Studies have revealed that human umbilical cord mesenchymal stem cell-derived exosomes (hucMSC-exosomes) can attenuate apoptosis in cardiomyocytes through the AMPK/mTOR signaling pathway, which alleviates the severity of viral myocarditis (Gu et al., 2020). Moreover, the proteasome inhibitor MG-132 suppresses the deterioration of cardiac function via the AMPK signaling pathway (Zhang et al., 2020). Consequently, we hypothesize that metformin behaves as an AMPK signaling pathway agonist and may attenuate myocardial damage in the acute and chronic phases of viral myocarditis. In the acute phase, excessive autophagosome formation induced by CVB3 in host cells requires phospholipids and cholesterol, whose accumulation can be suppressed by metformin (Xie et al., 2015). In addition, metformin can restore autophagic flux associated with lysosomal function. In the chronic phase of viral myocarditis, infection with CVB3 induces fibroblast proliferation and increases collagen secretion, leading to pathological myocardial remodeling and even heart failure. However, this condition can be attenuated by activating the AMPK signaling pathway (Jiang et al., 2016).

Resveratrol is an autophagy agonist that improves autophagic flux by activating the SIRT1/AMPK signaling pathway and enhancing the expression of TFEB, which is associated with lysosomal function. Resveratrol can activate Sirtuin 1 (SIRT1), which plays an important role in the deacetylation process of autophagy (Wang et al., 2009; Duan et al., 2016). Downregulation of SIRT1 by microRNA-34a exacerbates cardiomyocyte apoptosis in CVB3-induced viral myocarditis (Jiang et al., 2019). In contrast, activation of the SIRT1/AMPK signaling pathway by miR-217 or miR-543 reduces inflammation and myocardial injury in children with viral myocarditis (Xia et al., 2020). Resveratrol can act as a SIRT1 agonist and not only improve cardiac function and alleviate myocardial injury in acute viral myocarditis but also suppress collagen hyperplasia in chronic viral myocarditis (Wang et al., 2009). However, further studies are needed to determine whether resveratrol exerts this protective effect by directly regulating autophagy.

Rapamycin is an inhibitor of the mammalian target of rapamycin (mTOR) pathway that acts on multiple targets to regulate autophagy. On the one hand, when the nutrient supply is adequate, mTOR blocks the formation of ATG1-ATG13-ATG17, which plays an important role in the induction of autophagy by phosphorylating ATG13 excessively (Xiang et al., 2020). On the other hand, mTOR can directly phosphorylate TFEB, which is necessary for lysosomal biogenesis. Phosphorylated TFEB is present in the cytoplasm. This factor cannot translocate into the nucleus to induce the transcription of target genes (Napolitano and Ballabio, 2016). Studies have shown that rapamycin prevents cardiac fibrosis by reducing autophagic flux damage in transverse aortic constriction (TAC)-induced cardiac hypertrophy (Qu et al., 2021). Moreover, rapamycin inhibits the expression of Smad and the secretion of type I collagen, attenuating cardiac fibrosis induced by CVB3 (Chen et al., 2008). However, whether this effect is achieved by improving autophagy requires further study. In addition, further exploration of rapamycin treatment in viral myocarditis, including its treatment efficacy and application stage, is needed.

### 5.2 Application of Autophagy Inhibitors in Viral Myocarditis

CVB3 exploits the double-membrane structure of autophagosomes to undergo replication. Suppression of autophagosome formation during periods of CVB3 replication may attenuate damage to the host. Wortmannin and 3-MA are widely used autophagy inhibitors that suppress class III PI3K (Wu et al., 2010). It has been reported that wortmannin decreases CVB3 replication, leading to reductions in viral titer in cardiomyocytes, which is consistent with the effects of short hairpin RNA (shRNA) ATG5 lentiviral plasmids. These studies show that blocking autophagosome formation rather than a nonspecific effect of wortmannin reduces CVB3 replication (Zhou et al., 2015). In addition to its effect of decreasing viral titer, 3-MA can also reduce the levels of inflammatory factors, such as IL-6 and TNF-α, in cardiac fibroblasts (Shi et al., 2016; Zhai et al., 2016). A similar effect was observed with Atg7 siRNA, suggesting that 3-MA may achieve its anti-inflammatory effects by influencing autophagy during the early stages of viral infection (Zhai et al., 2016).

Chloroquine is a pH-neutralizing amine that preferentially accumulates in acidified organelles such as lysosomes and endosomes. This agent blocks membrane processes, including endosome/multivesicular body-to-lysosome fusion, as well as the fusion of these compartments with autophagosomes. It has been revealed that chloroquine inhibits host antiviral responses by attenuating the type I interferon signal transduction pathway mediated by Toll-like receptor 3 (TLR3), which may be related to its effect on virus antigen presentation by exacerbating autophagy lysosome damage (Gorbea et al., 2010).

Taken together, we found that autophagosome formation is increased, but autophagosome-lysosome fusion is blocked in viral myocarditis. Since CVB3 can exploit autophagosomes to proceed with their replication, the use of an inhibitor that acts on excessive autophagosome formation and an agonist targeting the repair of autophagosome-lysosome fusion may relieve the direct damage of the virus to the myocardium in the acute phase of viral myocarditis. In the chronic phase of viral myocarditis, excessive fibrosis may lead to pathological myocardial remodeling and even heart failure. The use of autophagy agonists that inhibit cardiac fibroblast proliferation and collagen synthesis may exert protective effects on chronic damage in viral myocarditis. In conclusion, targeting the different pathological stages in viral myocarditis is a key strategy in our future efforts.

## 6 Conclusion

In recent years, scientists have developed a deeper understanding of the pathogenesis of viral myocarditis. However, due to its diverse clinical outcomes, the diagnosis and treatment of viral myocarditis continue to be challenging. Therefore, the mechanism and treatments of viral myocarditis need to be further explored.

In this review, we discussed the roles of autophagy in the mechanism and disease progression of viral myocarditis and found that autophagy was deeply involved in the development of viral myocarditis. Typically, autophagy plays important roles in clearing viruses and activating the antiviral response in the body. However, CVB3 exploits the double-membrane structure of autophagosomes to proceed with replication within cardiomyocytes. Moreover, CVB3 increases autophagosome formation or suppresses the degradation of autophagosomes. Therefore, autophagy activation is not absolutely favorable for viral myocarditis therapy. Researchers should focus on therapies targeting steps in the process of autophagy that are affected by CVB3 to moderately inhibit autophagosome formation and alleviate lysosome impairment. In cardiac fibroblasts, autophagy activation promotes inflammatory cytokine production in viral myocarditis. Moreover, although the immune system profoundly affects the course of viral myocarditis, the roles of autophagy in the immune system have been poorly investigated, which represents an important future direction for research.

In viral myocarditis, there are still some inadequacies in autophagy studies. There have been few studies on the mechanisms of autophagy that are affected by CVB3 *in vivo*, and relevant studies have mostly been conducted on HeLa cells. Moreover, the transformation of mechanistic research toward clinical applications is further limited by the lack of associated clinical data.

In addition, the application of autophagy agonists and inhibitors in viral myocarditis has been discussed in terms of their different mechanisms of action. Personalized medication should be used according to the different stages of viral myocarditis. Specifically, further studies are needed to determine the type, dose, and timing of autophagy agonists and inhibitors when used to treat viral myocarditis.
